# N_2_-fixing bacteria are more sensitive to microtopography than nitrogen addition in degraded grassland

**DOI:** 10.3389/fmicb.2023.1240634

**Published:** 2023-09-15

**Authors:** Chengyi Li, Enrique Valencia, Yan Shi, Guiyao Zhou, Xilai Li

**Affiliations:** ^1^College of Agriculture and Animal Husbandry, Qinghai University, Xining, China; ^2^Departamento de Biodiversidad, Ecología y Evolución, Facultad de Ciencias Biológicas, Universidad Complutense de Madrid, Madrid, Spain; ^3^School of Environment, The University of Auckland, Auckland, New Zealand; ^4^German Centre for Integrative Biodiversity Research (iDiv) Halle-Jena-Leipzig, Leipzig, Germany; ^5^Institute of Biology, Leipzig University, Leipzig, Germany

**Keywords:** nitrogen addition, N_2_-fixing bacteria, alpine meadow, slope, Tibetan Plateau

## Abstract

**Introduction:**

Soil bacteria play a crucial role in the terrestrial nitrogen (N) cycle by fixing atmospheric N_2_, and this process is influenced by both biotic and abiotic factors. The diversity of N_2_-fixing bacteria (NFB) directly reflects the efficiency of soil N fixation, and the diversity of NFB in degraded alpine meadow soil may change with different N fertilizing levels and varied slopes. However, how N addition affects the diversity of NFB in degraded alpine meadows, and whether this influence varies with slope, remain poorly understood.

**Methods:**

We conducted an N addition field experiment at three levels (2, 5, and 10 g N·m^−2^·a^−1^) to study the effects of N addition on soil NFB diversity on two different slopes in a degraded meadow on the Tibetan Plateau.

**Results:**

There were significant differences in the dominant bacterial species between the two slopes. The Chao1 index, species richness, and beta diversity of NFB did not differ significantly between slopes, but the Shannon index did. Interestingly, N addition had no effect on the diversity of NFB or the abundance of dominant bacteria. However, we did observe a significant change in some low-abundance NFB. The community composition and diversity of NFB were significantly positively correlated with slope and soil physicochemical properties (e.g., total potassium, pH, and total nitrogen).

**Conclusions:**

Our study highlights the variation in NFB communities among different slopes in degraded alpine meadows and their resilience to exogenous N addition. Our results also underscore the importance of considering the effects of micro-topography on soil microbial communities in future studies of alpine ecosystems.

## Introduction

Soil nitrogen (N) is a major component of terrestrial ecosystem productivity (Xu et al., 2015). It is mostly fixed by plant growth in the soil, which makes the N supply capacity of soils a determining factor in plant growth (Nannipieri and Eldor, 2009); however, not all soil N can be absorbed by plants. In general, ammonium and nitrate are two effective N forms that can be directly absorbed by plants from the soil under the action of microorganisms (Wang et al., 2020). The microorganisms that can directly use atmospheric N are called N_2_-fixing microbes, and the conversion of N_2_ to NH_3_ mediated by N_2_-fixing microbes is called biological N fixation, which is the key source of available N in soils (Lindsay et al., 2010; Che et al., 2017). Actually, the N_2_-fixing microbes are composed of prokaryotes, including bacteria and archaea. Among them, N_2_-fixing bacteria (NFB) are characterized by the ferritin *nifH* gene with the catalytic function of nitrogenase (Zehr et al., 2003), which is a genetic marker that has been widely used to examine the community composition of NFB (Kang et al., 2013). Changes in the quantity and diversity of NFB can directly reflect the efficiency of soil N fixation and the normal operation of soil N cycling, thus, they are important indicators of soil functions that have been restored in different types of restored vegetation (Li et al., 2013). It is, therefore, of critical scientific importance to study the community composition and diversity of NFB.

Alpine meadows are a typical grassland ecosystem on the Qinghai-Tibet Plateau (QTP) (Gao et al., 2012). These communities are commonly distributed in highlands and mountainous areas (Zi et al., 2015) and are characterized by a fragile ecological environment and unique biogeochemical processes (Wischnewski et al., 2011). Hence, these areas are more sensitive to human disturbance and global environmental change (Lan, 2004; Wischnewski et al., 2011). In recent years, the alpine meadows on the QTP have suffered serious destruction; for instance, a large area of fine grasslands has been degraded to “*heitutan*” (bare land) (Sun et al., 2013), which is especially common in the Sanjiangyuan area of the central QTP (Chong and Zhang, 2015; Shao et al., 2017). These changes lead to an ecological imbalance in the QTP (Wang et al., 2021; Xu et al., 2021). Thus, effective measures must be taken to prevent the alpine meadow from being degraded.

One common prevention method is *via* N addition to improve soil productivity and maintain an ecological balance in the degraded grassland ecosystems (Wang et al., 2017). N addition promotes plant growth, modifies soil fertility, and induces soil acidification, which further changes soil microbial communities (Verburg et al., 2010; Tian and Niu, 2015; Chen et al., 2017; Han et al., 2017). This is because even small changes in the soil environment may cause a strong response in soil microorganisms that can quickly adapt to the new environment or make countermeasures (Geisseler et al., 2011; Fang et al., 2016). For example, the change in NFB is one of the most important indicators of soil degradation in degraded grasslands (Yan and Chen, 2001; Chen et al., 2020). He et al. (2010) found that exogenous N significantly affected NFB, modifying the soil microbial community composition and then soil N cycles. For instance, N fertilizers changed the composition of soil NFB and promoted the N_2_-fixing function of soil organisms while inhibiting the growth of NFB in grasslands (Orr et al., 2012; Parker, 2013). Numerous studies have been carried out to explore the effects of grazing, enclosure, supplementary sowing, and other management measures on soil properties. Nevertheless, how N addition affects NFB in degraded alpine meadows is still not fully understood. Moreover, due to the variety of terrain types in the Sanjiangyuan region, the structure and diversity of NFB may vary with the slopes of the grassland. However, few studies have explored how NFB change with slope. These knowledge gaps may hamper a better prediction of soil NFB feedback on future climate change.

Here, we conducted an N addition field experiment at three levels on two slopes in a degraded alpine meadow, assuming that N addition will change the community structure of soil NFB and reduce the diversity and that the response of soil NFB to N addition is different on different slopes. Specifically, the following two objectives were explored: (1) How does N addition affect soil NFB on different slopes in a degraded meadow? and (2) What are the main factors affecting NFB?

## Methods

### Field experiment description

**Figure 1:**
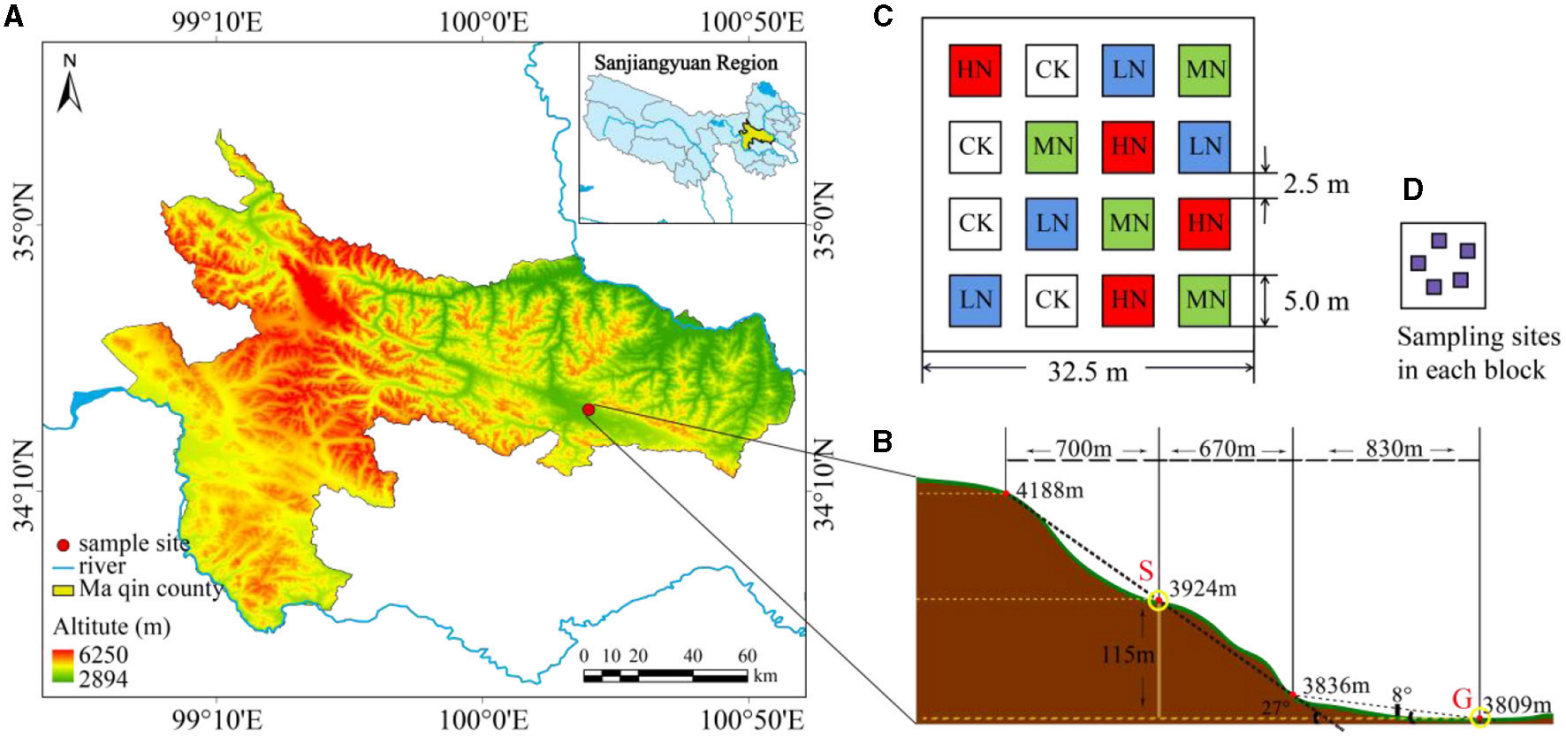
**(A)** Location of the study site. **(B)** The distribution of gentle slope area and steep slope area. **(C)** The distribution of fertilization plots. **(D)** Sampling sites in each block. CK, control plot; LN, low N addition; MN, medium N addition; HN, high N addition.

The study was conducted in Maqin County, Guoluo Tibetan Autonomous Prefecture, Qinghai Province, China. This site is situated at latitude 34°25′20.41″ N, longitude 100°19′55.72″ E (Figure 1A), with an average altitude of 3,768 m. The average annual precipitation and temperature were 528.8 mm and −3.9°C, respectively (Li et al., 2022a). The research site is a moderately degraded alpine grassland (Li et al., 2022b), and the soil type is alpine meadow soil (Zuo and Le, 1980). In addition, the grassland is grazed uncontrollably year-round. The main plant species on different slopes are similar and consist of *Kobresia pygmaea, Kobresia humilis, Elymus nutans, Ligularia virgaurea*, and *Poa pratensis*, among others.

According to the data on dynamic change characteristics of atmospheric N deposition in the alpine meadow ecosystem of the Qilian Mountain on the central QTP (Zhu et al., 2016) and the expectation of a continuous increase of N deposition in the future (Wang et al., 2019), our experiment had a fully randomized block design, with two slopes (G: gentle slope; and S: steep slope), four fertilization levels at 0, 2, 5, and 10 g N·m^−2^·a^−1^ (designated as CK, LN, MN, and HN, respectively), and four replications. In May 2019, 50 × 50 m degraded alpine meadow plots were selected on two slopes. Both sites have a slope of 12.3° from west to north, but the steep slope has a gradient of 27.0° and the gentle slope's gradient is 8.0° (Figure 1B). There were 16 quadrats (5 × 5 m) in each slope type, and each fertilization level was randomly allocated, resulting in a total of 32 permutations. The spacing between quadrats was set at 2.5 m (Figure 1C). After the plots were set up, they were fertilized with the N supplement (NH_4_NO_3_, grain loading, N content 35%) in dry conditions once a year in May 2019 and 2020.

### Soil sample collection and analysis

In August 2020, when herbage was growing vigorously, soil samples were collected from five random cores (top 0–10 cm with 3 cm inner diameter) in differently treated plots (Figure 1D). These five soil samples were pooled to obtain a representative sample per quadrat, resulting in 32 samples in total. The samples were sieved to remove impurities, and a quarter of each soil sample was reserved for microbial detection. The remaining soil samples were separated into two fractions, of which the first was kept at 4°C. The second fraction was air-dried for 2 weeks, ground, and sieved to be used for determining the soil properties using the same measurement methods and data as described by Li et al. (2022b).

### Microbial community analysis

The *nifH* gene fragments were amplified using primers *nifH*-F (AAAGGYGGWATCGGYAARTCCACCAC) and *nifH*-R (TTGTTSGCSGCRTACATSGCCATCAT). For more accurate analysis results, after the original sequencing data were launched, the optimized sequences were obtained through sequence splicing, filtering, and chimera removal to control the data quality. With a 97% similarity using Uparse (Edgar, 2013), the operational taxonomic units (OTUs) used were the Silva (Release128/132; http://www.arb-silva.de) database for clustering and annotation (Quast et al., 2012). Diversity analyses were carried out based on these results.

We calculated alpha diversity (Shannon diversity index, Chao1, and observed species number) using the formula developed by Li et al. (2020b). Additionally, the beta diversity of N_2_-fixing bacterial communities among samples was analyzed with non-metric multidimensional scaling (NMDS).

### Data statistics

The Kruskal test was carried out using the q-value package to analyze the different slopes as well as OTUs and the abundance differences of NFB in relation to N addition treatment for the same slope. If the *P*-value is <0.05, it is regarded as a different OTU or species.

The response of soil alpha diversity to different N application levels on the same slope was examined using a one-way ANOVA with Duncan *post-hoc* test. The Mantel test and Pearson test were used to evaluate the correlation among all these variables. The different indicators between slopes were analyzed by an independent sample *t*-test (α = 0.05).

A NetWork analysis was based on Spearman's test method, which selects the top 20 genus results of all samples for correlation analysis and takes the corresponding phylum as the legend. The calculated results filter out those whose correlation value |*R*| < 0.6 or whose *P*-value is >0.05 for drawing.

Species composition histogram and Venn diagram were plotted in the R language. All the analyses, including NMDS and network analysis, were conducted with R 4.1.2 using the vegan (Oksanen et al., 2013), ggplot2 (Wilke, 2019), psych (Revelle, 2010), ggcor (Huang et al., 2020), and dplyr (Wang, 2017) packages.

## Results

### Composition of N_2_-fixing bacterial community

**Figure 2:**
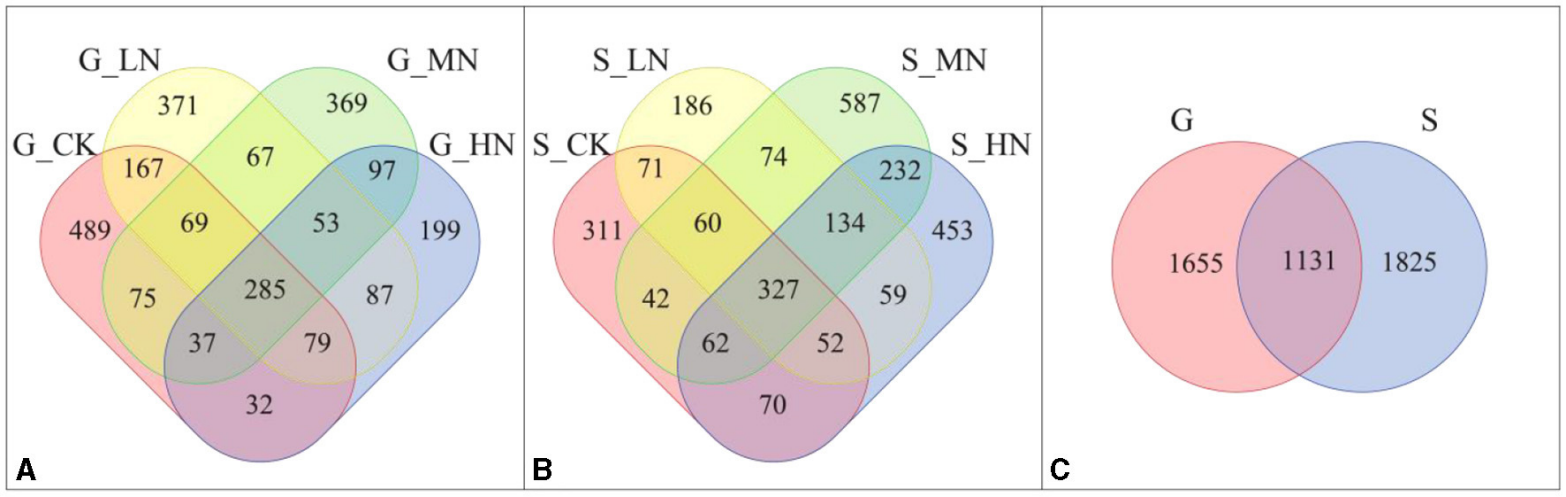
Venn diagrams of the different slopes and N addition levels **(A)** number of OTUs on the gentle slope; **(B)** number of OTUs on the steep slope; **(C)** total number of OTUs from both the gentle and the steep slope. G-CK refers to the control on the gentle slope; G-LN, G-MN, and G-HN refer to the N addition treatments at low, medium, and high levels on the gentle slope; S-CK refers to the control on the steep slope; S-LN, S-MN, and S-HN are the N addition treatment at low, medium, and high levels on the steep slope, respectively; G is the gentle slope; and S is the steep slope.

Different amounts of OTUs were obtained under different slopes and at different N addition levels (Figure 2). There were 30 and 22 OTUs with significant differences among the different N application levels on the gentle and steep slopes, respectively, and 472 OTUs with significant differences between the two slopes (*P* < 0.05; Supplementary Table S1).

**Figure 3:**
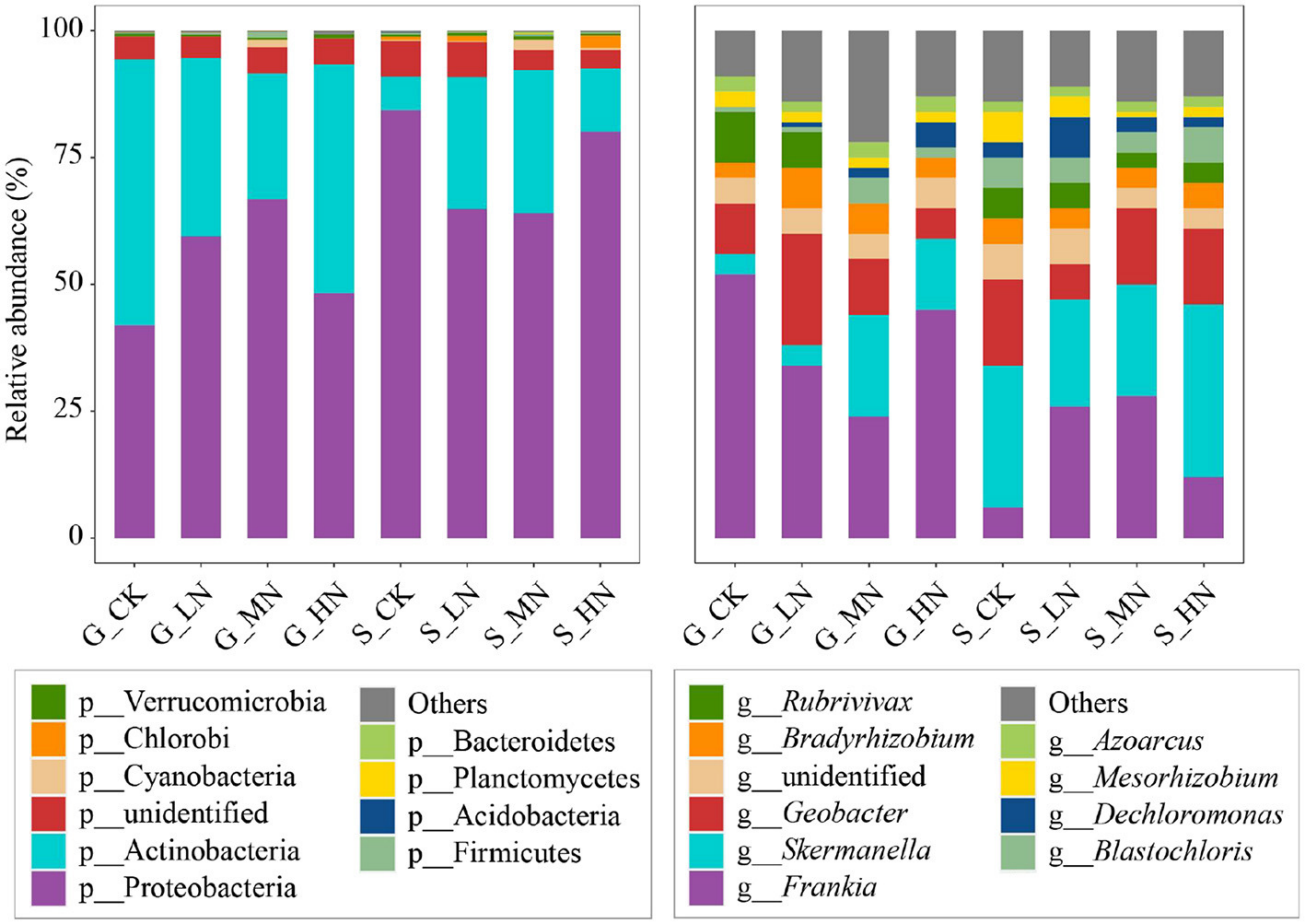
Composition of soil N_2_-fixing bacteria on the different slopes with different N addition levels at the phylum and the genus levels. The acronyms are described in Figure 2.

Proteobacteria and Actinobacteria were the dominant phyla of NFB (Figure 3), and their abundance had no significant difference on the same slope to which N was added (Supplementary Table S2). Significant differences existed between both slopes in dominant bacteria, including Proteobacteria, Actinobacteria, and some NFB of very low abundance (such as Chlorophyta and Bacteroidetes). Actinobacteria were significantly higher on the gentle slope than on the steep slope; in contrast, Proteobacteria on the gentle slope were significantly lower (*P* < 0.05).

The dominant NFB genera identified were *Frankia, Skermanella*, and *Geobacter* (Figure 3). The first one belongs to Actinobacteria, and the latter two belong to Proteobacteria. The abundance of the dominant genera of NFB on the same slope and the different N additions were not significant, but significant differences existed in some low-abundance NFB (relative abundance <0.5%; *P* < 0.05). For example, there were differences in N addition in *Brachybacterium, Actinoplanes, Bosea, Janthinobacterium, Plantibacter, Alterrythrobacter*, and *Curvibacter* on the gentle slope and in *Symploca, Achromobacter, lusitaniella, Gluconacetobacter*, and *Roseomonas* on the steep slope. *Brachybacterium* was selected on the gentle slope; *Symploca* and *Achromobacter* were selected on the steep slope (relative abundance >0.1%) (Supplementary Table S3). Compared with CK, HN significantly reduced *Brachybacterium* on the gentle slope (*P* < 0.05; Supplementary Table S4). Compared with CK, *Achromobacter* on the steep slope significantly decreased under MN (*P* < 0.05), while *Symploca* significantly increased under HN (*P* < 0.05). There were significant differences between the two slopes in 59 genera, including *Frankia* and *Skermanella* (*P* < 0.05; Supplementary Table S5).

**Figure 4:**
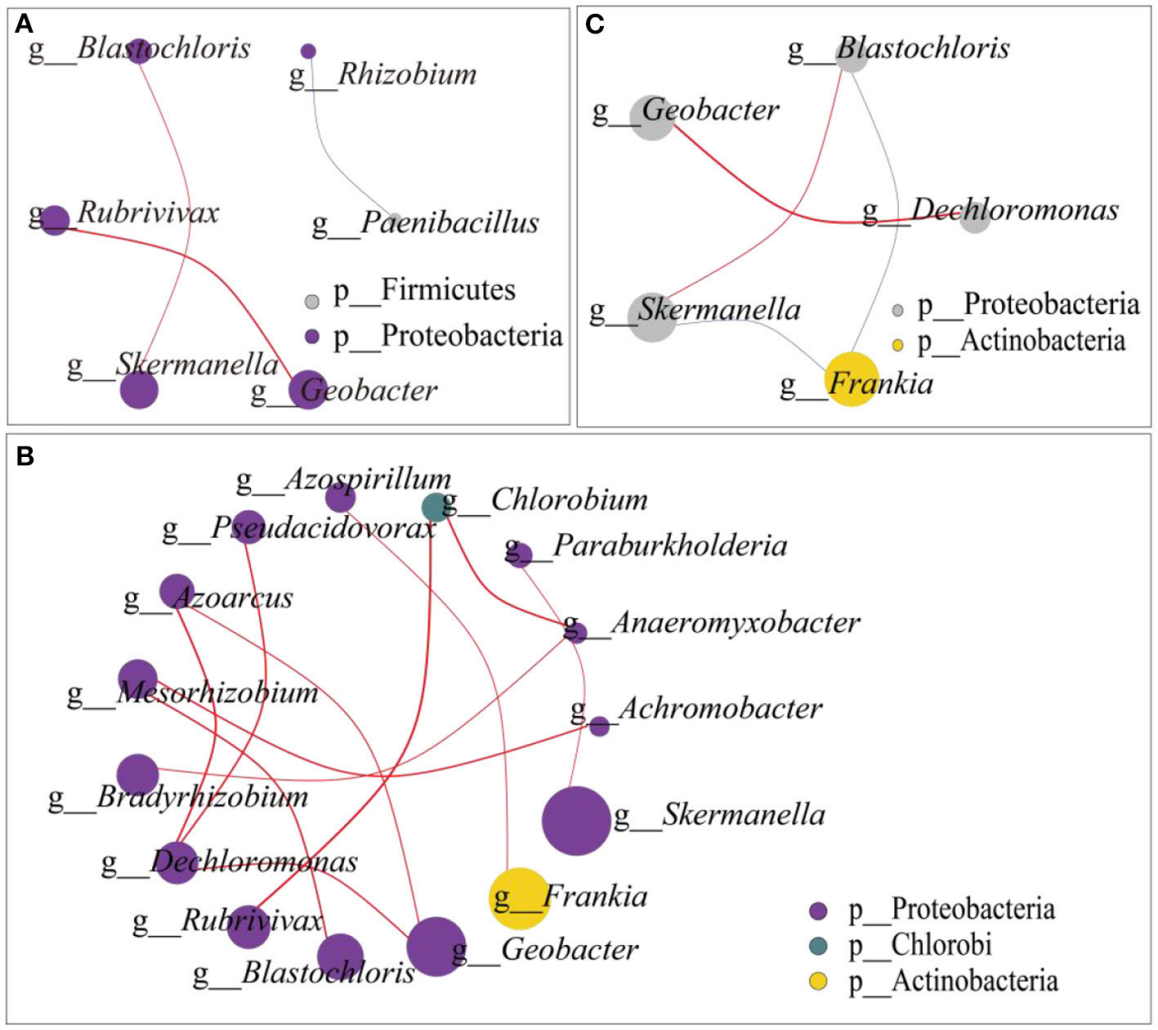
Microbial network interaction of N_2_-fixing bacteria **(A)** microbial network interaction on the gentle slope; **(B)** microbial network interaction on the steep slopes; **(C)** microbial network interaction from both the gentle and the steep slopes. The dot size in the graph represents the abundance, and the line thickness represents the correlation. The dot color represents the door to which it belongs, while the red line represents a positive correlation, and the blue line represents a negative correlation.

On the gentle slope (Figure 4A), *Paenibacillus* belongs to Firmicutes, and the rest belongs to Proteobacteria. The first part was composed of *Blastochloris, Rubrivivax, Skermanella*, and *Geobacter*, which were positively correlated. In the second part, *Rhizobium* and *Paenibacillus* were negatively correlated. On the steep slope (Figure 4B), *Chlorobium* belongs to the Chlorobi phylum, *Frankia* belongs to the Actinobacteria phylum, and the other 13 genera belong to the Proteobacteria phylum. Among them, *Achromobacter*, which showed significant differences among different N addition levels, was positively correlated with *Mesorhizobium*. The network interaction map of the two slopes showed that the dominant genera *Frankia* was negatively correlated with *Skermanella* and *Blastochloris* (Figure 4C).

### Diversity of soil N_2_-fixing bacteria

**Figure 5:**
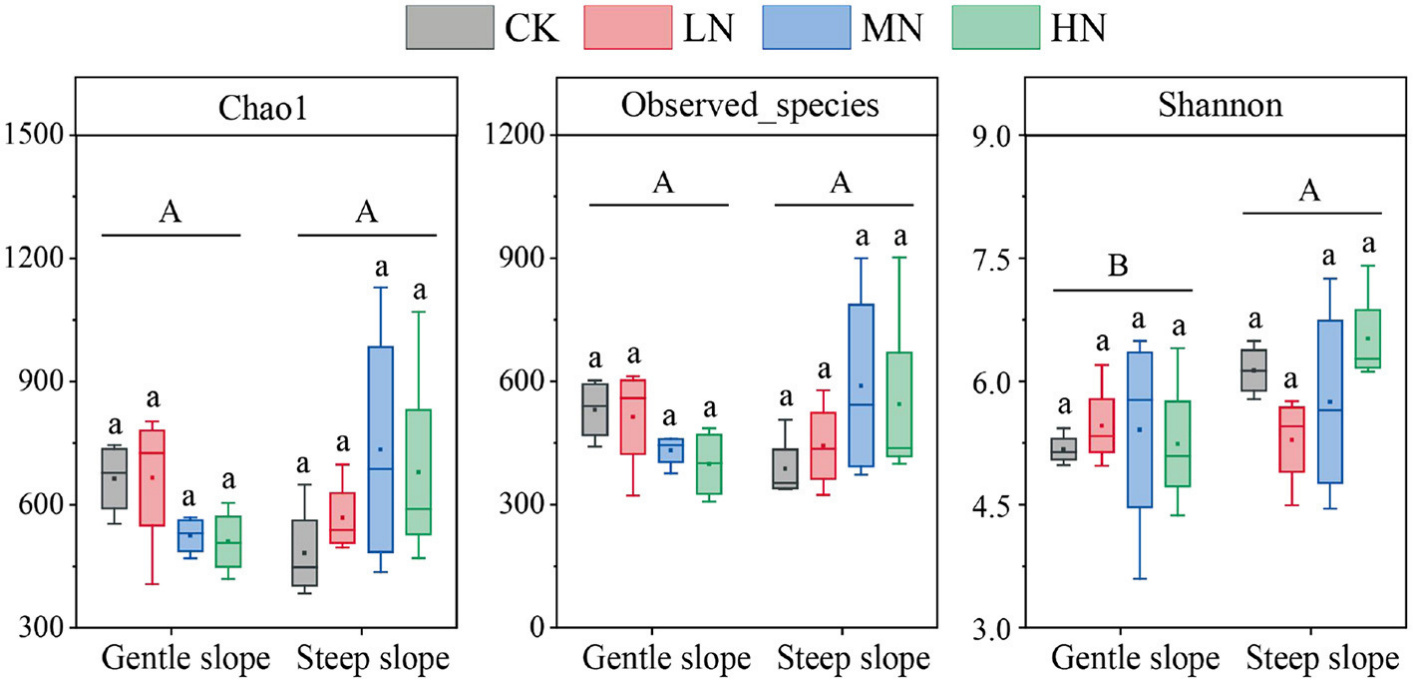
Abundance and diversity index of N_2_-fixing bacteria in soil samples. Different lowercase letters represent significant differences among N addition levels in the same slope, and different uppercase letters represent significant differences between the slopes. The acronyms are described in Figure 2.

**Figure 6:**
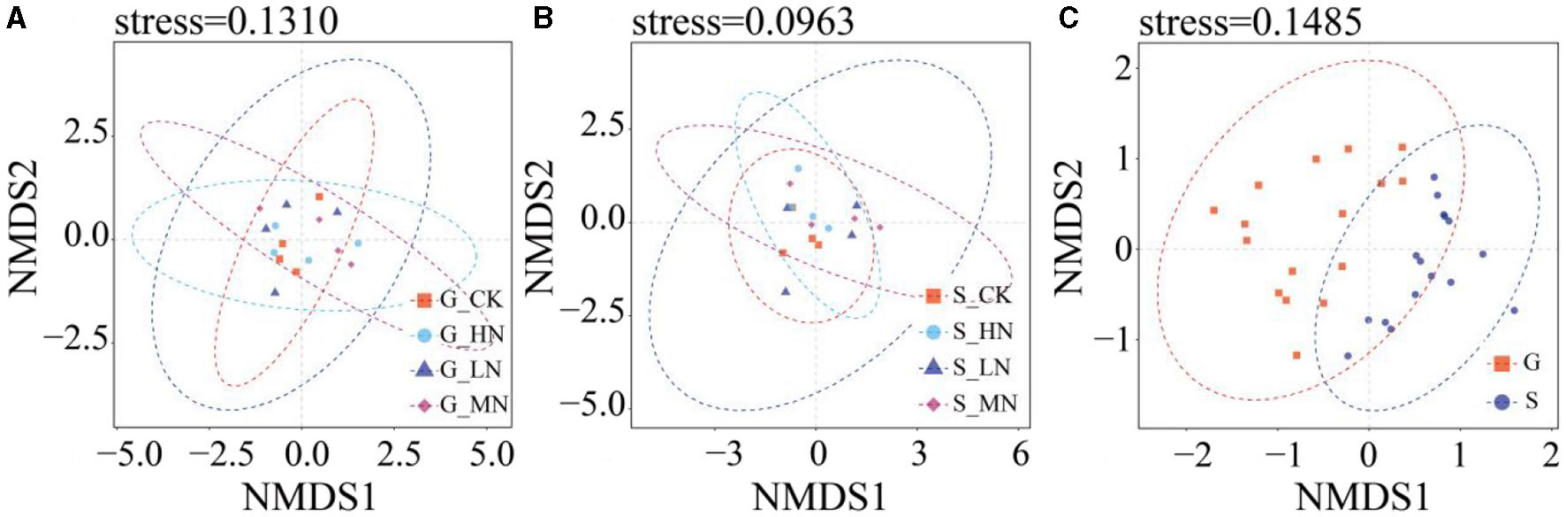
NMDS analysis of OTU level **(A)** groups of N addition on the gentle slope; **(B)** groups of N addition on the steep slope; **(C)** groups from both the gentle and the steep slopes.

On the same slope, the alpha diversity of soil NFB did not differ significantly with N addition levels (Figure 5). The Chao1 and the number of species observed did not differ significantly between the slopes, but the Shannon index did (*P* < 0.05). At the same time, the NMDS showed no differences among N addition levels on the same slope and between the different slopes (Figure 6). There were significant differences in NFB community diversity in CK between the slopes (*P* < 0.05; Supplementary Table S6).

### Relationship between soil properties and N_2_-fixing bacteria

**Figure 7:**
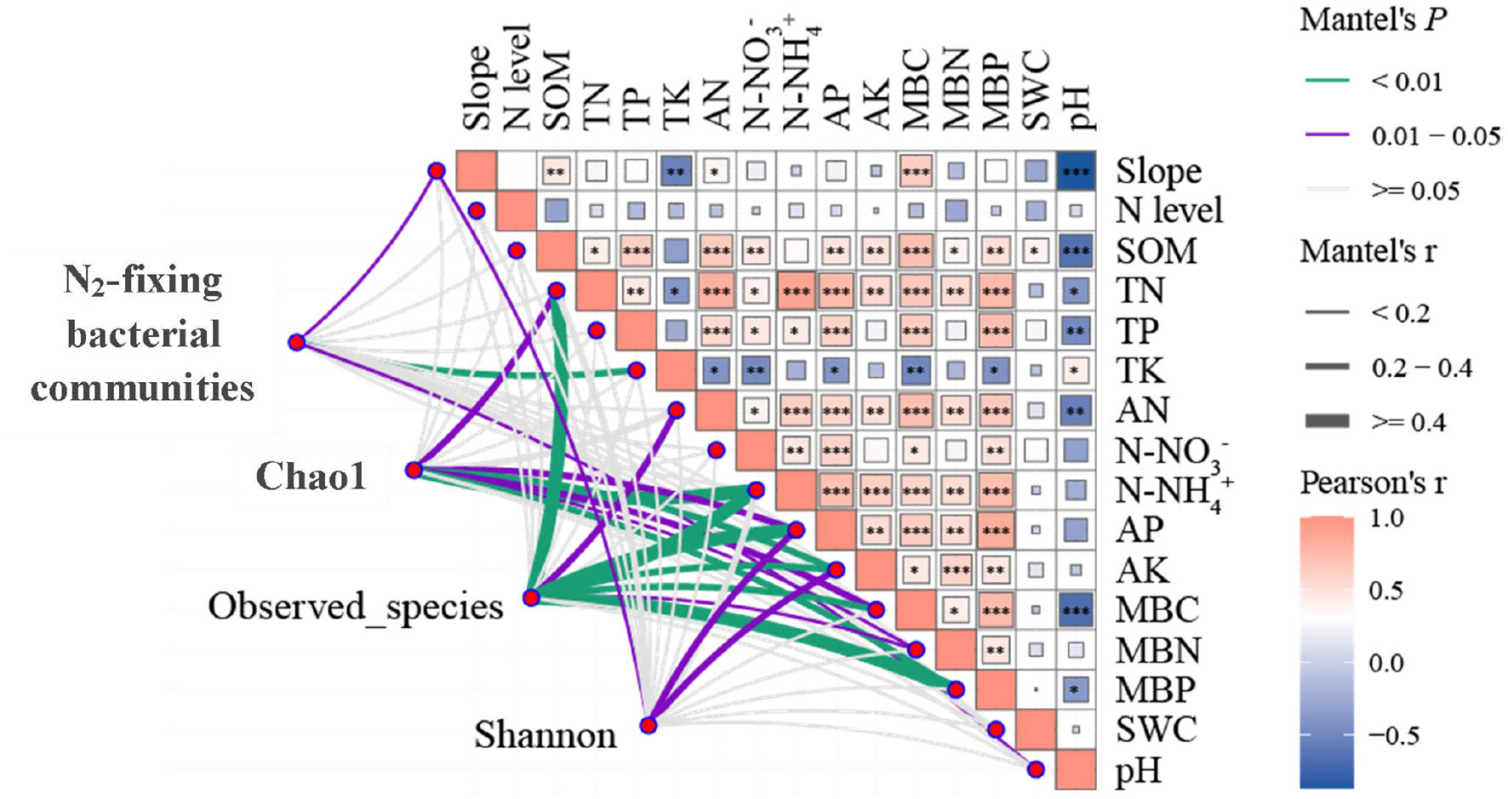
Correlation of N_2_-fixing microbial composition and diversity with slope, N addition levels, soil physicochemical properties, and microbial biomass. Mantel edge width corresponds to the Mantel r value, and edge color indicates statistical significance. The color gradient of Pearson correlation coefficient r represents the paired correlation of variables. 27.0° for the steep slope and 8.0° for the gentle slope. N addition levels include CK: 0 g N·m^−2^; LN: 2 g N·m^−2^; MN: 5 g N·m^−2^; and HN: 10 g N·m^−2^. N_2_-fixing microbial community includes N_2_-fixing bacteria phylum with significant differences among different slopes (Chlorobi, Chlorophyta, Actinobacteria, Proteobacteria, Euryarchaeota, and Bacteroidetes). ^*^indicates 0.01 < *P* < 0.05, ^**^indicates *P* < 0.01, ^***^ indicates *P* < 0.001.

A significantly positive correlation was found among slope, TK, pH, and community abundance of NFB (*P* < 0.05; Figure 7). Furthermore, TN, N-NH${}_{4}^{+}$, AP, AK, MBC, MBN, MBP, and Chao1 were significantly and positively correlated (*P* < 0.05). A significantly positive correlation was also found among TN, AN, N-NH${}_{4}^{+}$, AP, AK, MBC, MBN, MBP, and observation species (*P* < 0.05). In addition, a significantly positive correlation was found among slope, AP, AK, and Shannon (*P* < 0.05).

**Figure 8:**
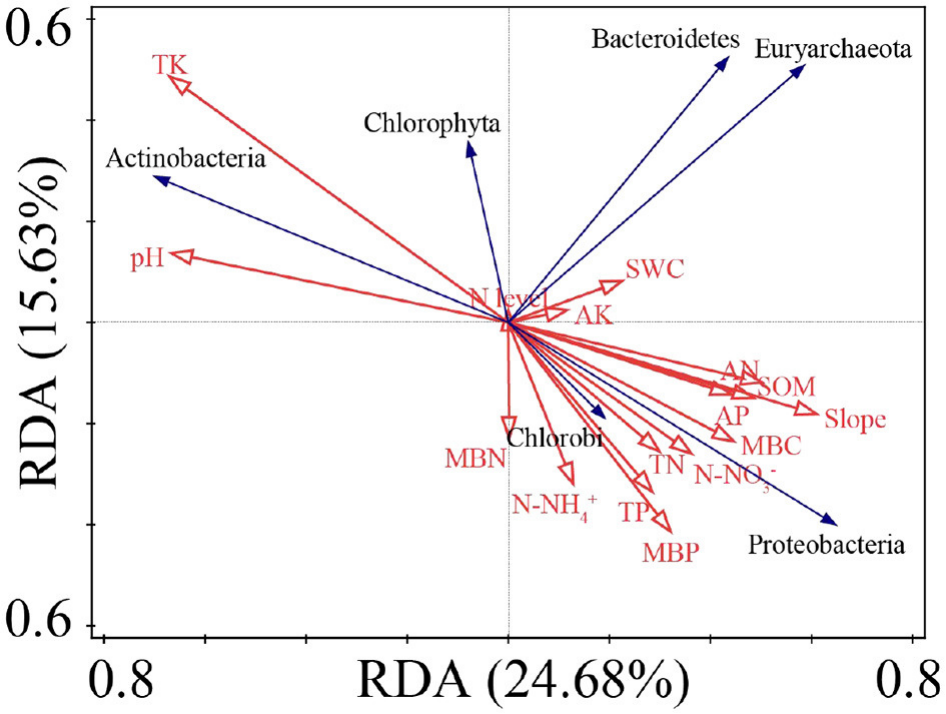
RDA ranking of soil physicochemical indexes and N_2_-fixing bacteria abundance with significant differences.

The ranking results of the redundancy analysis (RDA) showed that the first and second ranking axes accounted for 24.68% and 15.63% of the total species variability, respectively (Figure 8). TK, pH, and slope account for 14.9%, 12.0%, and 10.6% of variation in the prime index (Supplementary Table S7), respectively.

## Discussion

N addition showed no significant effect on the dominant genera of NFB. Nonetheless, it had a significant effect on the rare genera of NFB; besides, the response of NFB in degraded alpine meadows to N application on different slopes was different. The sensitivity of all NFB phyla to N application was weakened because of the low abundance of genera with significant differences in NFB communities; this indicated that the species specificity of soil NFB under different N addition levels was weak in degraded alpine meadows. At the same time, N addition had no significant effect on the richness and diversity of NFB, regardless of the slopes. This finding totally contravenes that reported by Qin (2021). They reported that the abundance and diversity of NFB changed significantly after N addition such as N addition significantly changed *Azorhizobium* and *Nostoc* (Qin, 2021). Meanwhile, the Shannon index of NFB of 15 g N·m^−2^·a^−1^ decreased significantly when compared with 1.5 g N·m^−2^·a^−1^, but the number of observed species and the Chao1 of *nifH* bacteria did not respond to N addition (Qin, 2021). Compared with CK, no significant difference in the Shannon index of NFB existed among 1.5, 3, 5, and 10 g N·m^−2^·a^−1^ (Qin, 2021). Previous studies had also shown that the long-term application of N fertilizer reduced the competitiveness of soil NFB, which changed their diversity and community structure (Mirza et al., 2014; Wang et al., 2016). Some of these differences may be attributed to different types of grasslands with different plant community compositions, species characteristics, and external environmental resources. For example, different plant species may promote different absorptions and utilizations of soil N due to their different growth types (Weintraub, 2004) and periods of growth and development. In addition, it should be mentioned that there are also differences in the availability of N sources and in the content of available N in the soil (Hodge et al., 2000). Besides, there may also be differences in the composition of the bacterial population structure containing specific functional genes that lead to transformation into N. Moreover, our results may also depend on the setting of the N addition level and on the duration of the N addition.

As the main topographic factor, slope has an important impact on grassland soil water content, soil erosion degree, and soil thickness, resulting in the differentiation of soil nutrients on different slopes (Zhang, 2019). Many studies have already shown that both the structure and composition of NFB were affected by the physical and chemical environment of the soil (Li et al., 2013). Comparing CK on both slopes, the content of SOM and AN after N addition was significantly higher on the steep slope, while the opposite was true for pH. N addition can also stimulate the change in the composition of NFB on the two slopes. The difference in the dominant phylum bacteria, such as Actinobacteria and Proteobacteria, changed with the slopes. These changes may be related to the natural TK of the gentle and steep slopes. Our study found that there were significant differences in TK between CK on the two slopes and a significant positive correlation between TK and the composition of NFB. We suggest that the difference in the dominant communities of NFB was limited by the total potassium in the soil. This may be because the total potassium content in soil restricts the growth of plants or the physiological metabolism of NFB, such as cell wall synthesis and cell division, which further affects the growth and activities of NFB. In addition, total potassium can also affect the growth environment of soil microorganisms and change the microbial community structure, thus affecting the growth and activities of NFB. Therefore, the response of NFB to an exogenous N addition was not consistent in alpine meadows with different slopes. These results may be more related to the diversity differences of NFB under the natural conditions of gentle and steep slopes: the higher the NFB diversity index, the more stable the grassland soil environment (Zhao et al., 2018). Different levels of N addition will not change the richness and diversity of NFB. The results of our research on fertilization for 1 year showed that, in restoration, the positive effect of short-term exogenous N addition depends on slope, which was more suitable for the restoration of vegetation and soil bacterial diversity on the gentle slope. For the restoration of moderately degraded alpine meadows on the gentle slope, the N addition level should be controlled at ~10 g·m^−2^ (Li et al., 2020a,b, 2022b). However, it should be noted that the addition of N on the gentle slope will reduce the content of soil TK and increase the content of AP, while the addition of a high amount of N will reduce the content of soil TK and MBP. It should be noted that the difference in altitude between the two slope plots may also be one of the reasons for the observed differences in experimental results, and this will be further verified in future studies. In addition, the potential N_2_ fixation rate (or nitrogenase activity) in the soil will be measured to further demonstrate the effect of N addition on the soil's N_2_-fixing bacterial community.

## Conclusion

N addition on the same slope had no significant effect on the richness and diversity of NFB, but significant differences in the richness and diversity of NFB existed between two slopes in a degraded meadow. These differences may be related to differences in the availability of phosphorus and potassium. It is not recommended to take measures of N addition to restore degraded alpine meadows on a steep slope. Our study showed the impact of topographic factors on degraded grasslands, highlighting the need to consider this information to better restore those degraded grasslands.

## Data availability statement

The original contributions presented in the study are included in the article/Supplementary material, further inquiries can be directed to the corresponding author.

## Author contributions

Field experiments were carried out by XL and CL. Data analysis was carried out by CL and YS. The manuscript was prepared by CL, with contributions from XL, GZ, EV, and YS. All authors contributed to the article and approved the submitted version.
