# Supplementary material for: N2-fixing bacteria are more sensitive to microtopography than nitrogen addition in degraded grassland
Source: Front Microbiol. 2023 Sep 15;14:1240634. doi: 10.3389/fmicb.2023.1240634 (PMC10540685; doi:10.3389/fmicb.2023.1240634)
Supplement: Supplementary file 1 [file Table_1.DOCX]

1. **fixing bacteria is more sensitive to microtopography than nitrogen addition in degraded grassland**

Chengyi Li^1^, Enrique Valencia^2^, Yan Shi^3^, Guiyao Zhou^4,5^, Xilai Li^1^*

*^1^College of Agriculture and Animal Husbandry, Qinghai University, Xining 810016, China.*

*^2^Departamento de Biodiversidad, Ecología y Evolución, Facultad de Ciencias Biológicas, Universidad Complutense de Madrid, Spain.*

*^3^School of Environment, the University of Auckland, Auckland 1010, New Zealand.*

*^4^German Centre for Integrative Biodiversity Research (iDiv) Halle-Jena-Leipzig, Puschstrasse 4, 04103 Leipzig, Germany.*

*^5^Institute of Biology, Leipzig University, Puschstrasse 4, 04103 Leipzig, Germany.*

*** Corresponding author**

Xilai Li

College of Agriculture and Animal Husbandry

Qinghai University, Xining 810016, China.

E-mail: [xilai-li@163.com](mailto:xilai-li@163.com)

**Supplementary Material**

**1.1 Supplementary Tables**

**Supplementary Table 1** Kruskal test of OTU species with significant differences

|  | OTU species |
| --- | --- |
| Different N addition treatments on gentle slope | OTU_1072, OTU_1194, OTU_1248, OTU_1258, OTU_126, OTU_1284, OTU_1418, OTU_1892, OTU_1902, OTU_1982, OTU_2184, OTU_225, OTU_2287, OTU_2300, OTU_2411, OTU_2568, OTU_307, OTU_31, OTU_347, OTU_348, OTU_488, OTU_59, OTU_625, OTU_738, OTU_747, OTU_749, OTU_765, OTU_803, OTU_925, OTU_937. |
| Different N addition treatments on steep slope | OTU_1209, OTU_1240, OTU_1619, OTU_1738, OTU_1843, OTU_2097, OTU_2143, OTU_2421, OTU_252, OTU_343, OTU_37, OTU_431, OTU_527, OTU_688, OTU_746, OTU_768, OTU_818, OTU_877, OTU_884, OTU_935, OTU_936, OTU_944. |
| Different slopes | OTU_1019, OTU_1022, OTU_1047, OTU_1069, OTU_1080, OTU_1082, OTU_1083, OTU_1084, OTU_1129, OTU_1132, OTU_1192, OTU_1198, OTU_1199, OTU_1205, OTU_1209, OTU_1239, OTU_1247, OTU_1255, OTU_1277, OTU_1286, OTU_1290, OTU_1291, OTU_1294, OTU_1296, OTU_1298, OTU_1300, OTU_1302, OTU_1307, OTU_1309, OTU_1310, OTU_1313, OTU_1314, OTU_1315, OTU_1316, OTU_1318, OTU_1319, OTU_1320, OTU_1321, OTU_1325, OTU_1328, OTU_1329, OTU_1330, OTU_1331, OTU_1333, OTU_1336, OTU_1337, OTU_1339, OTU_1342, OTU_1345, OTU_1346, OTU_1347, OTU_1349, OTU_135, OTU_1353, OTU_1354, OTU_1359, OTU_1360, OTU_1364, OTU_1365, OTU_1374, OTU_1376, OTU_1378, OTU_1379, OTU_1380, OTU_1382, OTU_1384, OTU_1385, OTU_1388, OTU_1390, OTU_1391, OTU_1396, OTU_1398, OTU_1406, OTU_1410, OTU_1411, OTU_1413, OTU_1415, OTU_1416, OTU_1417, OTU_1418, OTU_142, OTU_1425, OTU_1429, OTU_1432, OTU_1443, OTU_145, OTU_1455, OTU_1458, OTU_146, OTU_1460, OTU_1463, OTU_1469, OTU_1470, OTU_1472, OTU_1474, OTU_1475, OTU_1476, OTU_1483, OTU_1489, OTU_1498, OTU_1503, OTU_1527, OTU_1541, OTU_1546, OTU_1555, OTU_1570, OTU_1577, OTU_1583, OTU_1589, OTU_1605, OTU_1612, OTU_1621, OTU_1626, OTU_1642, OTU_1647, OTU_1653, OTU_1655, OTU_1659, OTU_1662, OTU_1665, OTU_1672, OTU_1698, OTU_1700, OTU_1702, OTU_1703, OTU_1704, OTU_1705, OTU_1711, OTU_1713, OTU_1714, OTU_1717, OTU_1720, OTU_1721, OTU_1723, OTU_1724, OTU_1726, OTU_1728, OTU_1735, OTU_1737, OTU_1738, OTU_1739, OTU_1741, OTU_1745, OTU_1746, OTU_1747, OTU_1754, OTU_177, OTU_1772, OTU_1773, OTU_1774, OTU_1778, OTU_1795, OTU_18, OTU_1816, OTU_1821, OTU_1832, OTU_1833, OTU_1834, OTU_1842, OTU_1846, OTU_1848, OTU_186, OTU_1860, OTU_1862, OTU_1865, OTU_1867, OTU_1869, OTU_1870, OTU_1872, OTU_1876, OTU_1877, OTU_1880, OTU_1888, OTU_1893, OTU_1900, OTU_1902, OTU_1904, OTU_1908, OTU_1909, OTU_1912, OTU_1916, OTU_1918, OTU_1922, OTU_1928, OTU_1930, OTU_1931, OTU_1933, OTU_1934, OTU_1935, OTU_1943, OTU_1947, OTU_1957, OTU_1963, OTU_1965, OTU_1991, OTU_20, OTU_2017, OTU_2025, OTU_2026, OTU_2043, OTU_2055, OTU_2057, OTU_2060, OTU_2061, OTU_2062, OTU_2113, OTU_213, OTU_2130, OTU_216, OTU_2196, OTU_2229, OTU_2289, OTU_234, OTU_236, OTU_2487, OTU_2488, OTU_250, OTU_2501, OTU_2510, OTU_2549, OTU_2564, OTU_266, OTU_2660, OTU_2698, OTU_270, OTU_2712, OTU_2732, OTU_2754, OTU_2792, OTU_2794, OTU_2840, OTU_2855, OTU_2882, OTU_2897, OTU_290, OTU_2902, OTU_2920, OTU_2972, OTU_2975, OTU_3017, OTU_3089, OTU_3121, OTU_313, OTU_3139, OTU_3176, OTU_3177, OTU_3178, OTU_3179, OTU_3184, OTU_3188, OTU_3192, OTU_3193, OTU_3203, OTU_321, OTU_3216, OTU_3259, OTU_326, OTU_3260, OTU_3287, OTU_3294, OTU_3295, OTU_3297, OTU_3318, OTU_3368, OTU_3379, OTU_339, OTU_3390, OTU_3391, OTU_3392, OTU_34, OTU_3408, OTU_3453, OTU_3463, OTU_3508, OTU_3553, OTU_3578, OTU_3581, OTU_3583, OTU_3584, OTU_3589, OTU_362, OTU_3626, OTU_3638, OTU_366, OTU_3690, OTU_370, OTU_3715, OTU_3722, OTU_3726, OTU_3727, OTU_3728, OTU_3729, OTU_373, OTU_3734, OTU_3735, OTU_3741, OTU_3755, OTU_3804, OTU_3807, OTU_3832, OTU_3833, OTU_3836, OTU_3837, OTU_3839, OTU_3873, OTU_3891, OTU_3901, OTU_3917, OTU_3924, OTU_393, OTU_3979, OTU_4, OTU_4044, OTU_4045, OTU_4046, OTU_407, OTU_4088, OTU_4091, OTU_4092, OTU_4094, OTU_4096, OTU_4097, OTU_4099, OTU_4101, OTU_4105, OTU_4106, OTU_4108, OTU_4111, OTU_4113, OTU_4115, OTU_4125, OTU_4133, OTU_4134, OTU_4148, OTU_415, OTU_4152, OTU_4156, OTU_4166, OTU_4168, OTU_4196, OTU_4199, OTU_42, OTU_4235, OTU_4266, OTU_4287, OTU_4317, OTU_4319, OTU_4369, OTU_4371, OTU_4448, OTU_4508, OTU_4509, OTU_4512, OTU_4514, OTU_4516, OTU_4517, OTU_4522, OTU_4571, OTU_4580, OTU_466, OTU_4676, OTU_4677, OTU_4684, OTU_4690, OTU_47, OTU_470, OTU_4713, OTU_4760, OTU_4798, OTU_4807, OTU_4808, OTU_4812, OTU_4836, OTU_493, OTU_499, OTU_505, OTU_51, OTU_511, OTU_52, OTU_527, OTU_530, OTU_531, OTU_532, OTU_536, OTU_538, OTU_540, OTU_543, OTU_544, OTU_545, OTU_55, OTU_554, OTU_555, OTU_560, OTU_561, OTU_563, OTU_564, OTU_568, OTU_572, OTU_576, OTU_578, OTU_584, OTU_586, OTU_591, OTU_6, OTU_60, OTU_600, OTU_601, OTU_604, OTU_607, OTU_608, OTU_613, OTU_614, OTU_615, OTU_618, OTU_619, OTU_621, OTU_622, OTU_624, OTU_625, OTU_627, OTU_63, OTU_630, OTU_636, OTU_639, OTU_64, OTU_642, OTU_643, OTU_644, OTU_649, OTU_655, OTU_663, OTU_665, OTU_67, OTU_680, OTU_684, OTU_685, OTU_689, OTU_695, OTU_699, OTU_701, OTU_702, OTU_704, OTU_705, OTU_71, OTU_715, OTU_716, OTU_719, OTU_725, OTU_730, OTU_738, OTU_743, OTU_758, OTU_777, OTU_779, OTU_782, OTU_793, OTU_801, OTU_816, OTU_823, OTU_837, OTU_844, OTU_858, OTU_866, OTU_867, OTU_868, OTU_871, OTU_883, OTU_888, OTU_901, OTU_93, OTU_943, OTU_976. |

**Supplementary Table 2** Kruskal test results of N-fixing bacteria phylum level

| Azotobacter phylum | *P*-value | | |
| --- | --- | --- | --- |
|  | G (N addition) | S (N addition) | G vs S |
| Chordata | 0.09 | 0.39 | 0.55 |
| Cyanobacteria | 0.20 | 0.48 | 0.05 |
| Chlorophyta | 0.23 | 0.69 | 0.00 |
| Candidatus_Peregrinibacteria | 0.28 | 0.24 | 1.00 |
| Chloroflexi | 0.29 | 0.66 | 0.79 |
| Firmicutes | 0.30 | 0.42 | 0.08 |
| Nitrospirae | 0.35 | 0.39 | 0.06 |
| Deinococcus-Thermus | 0.36 | 0.25 | 0.20 |
| Ascomycota | 0.37 | - | 0.40 |
| Thaumarchaeota | 0.39 | - | - |
| Chlorobi | 0.39 | 0.57 | 0.00 |
| Euryarchaeota | 0.39 | 0.18 | 0.02 |
| Arthropoda | 0.39 | - | - |
| Mucoromycota | 0.39 | - | 0.55 |
| Proteobacteria | 0.44 | 0.34 | 0.02 |
| Bacteroidetes | 0.49 | 0.76 | 0.02 |
| Planctomycetes | 0.49 | 0.73 | 0.17 |
| Acidobacteria | 0.52 | 0.88 | 0.97 |
| Basidiomycota | 0.54 | - | 0.97 |
| Streptophyta | 0.54 | - | 0.07 |
| Actinobacteria | 0.56 | 0.57 | 0.02 |
| unidentified | 0.56 | 0.47 | 0.50 |
| Gemmatimonadetes | 0.60 | 0.65 | 0.80 |
| Verrucomicrobia | 0.65 | 0.94 | 0.84 |
| Armatimonadetes | - | 0.09 | 0.15 |
| Spirochaetes | - | 0.20 | 0.15 |
| Haptista | - | 0.39 | 0.32 |

**Supplementary Table 3** N-fixing bacteria with significant differences in relative abundance of N addition at different slopes (relative abundance > 0.1%)

| Slope | Azotobacter genus | Relative abundance (%) | | | |
| --- | --- | --- | --- | --- | --- |
|  |  | CK | LN | MN | HN |
| Gentle slope | *Brachybacterium* | 0.48±0.18 | 0.16±0.04 | 0.28±0.48 | 0.03±0.04 |
| Steep slope | *Achromobacter* | 0.35±0.24 | 0.74±1.44 | 0.00±0.00 | 0.06±0.11 |
|  | *Symploca* | 0.00±0.01 | 0.00±0.01 | 1.29±2.58 | 0.21±0.22 |

**Supplementary Table 4** Multiple comparison of single factor AVOVA of Kruskal with different slope N addition treatments

| Slope | Azotobacter genus | *P*-value | | | | | |
| --- | --- | --- | --- | --- | --- | --- | --- |
|  |  | CK vs LN | CK vs MN | CK vs HN | LN vs MN | LN vs HN | MN vs HN |
| Gentle slope | *Brachybacterium* | 0.234 | 0.074 | 0.003 | 0.552 | 0.074 | 0.234 |
| Steep slope | *Achromobacter* | 0.410 | 0.003 | 0.116 | 0.030 | 0.454 | 0.155 |
|  | *Symploca* | 1.000 | 0.072 | 0.005 | 0.072 | 0.005 | 0.326 |

**Supplementary Table 5** Relative abundance of dominant N-fixing bacteria in different slopes and Kruskal test results

| Dominant bacteria of azotobacter | Relative abundance (%) | | *P*-value |
| --- | --- | --- | --- |
|  | Gentle slope | Steep slope |  |
| *Frankia* | 38.61±27.77 | 17.68±20.63 | 0.013 |
| *Skermanella* | 10.58±12.76 | 26.30±18.50 | 0.003 |

**Supplementary Table 6** Results of independent sample t test on soil physical and chemical properties, microbial biomass, N-fixing microbial community structure and diversity of degraded alpine meadow with different slopes in natural conditions

| Index | Variable | G-CK vs S-CK | |
| --- | --- | --- | --- |
|  |  | *F*-value | *P*-value |
| Soil physicochemical properties and microbial biomass | SOM | 0.054 | 0.340 |
|  | TN | 0.164 | 0.844 |
|  | TP | 0.096 | 0.740 |
|  | TK | 0.319 | 0.000 |
|  | AN | 0.149 | 0.564 |
|  | N-NO_3_^-^ | 0.971 | 0.387 |
|  | N-NH_4_^+^ | 4.298 | 0.633 |
|  | AP | 1.430 | 0.441 |
|  | AK | 14.931 | 0.606 |
|  | MBC | 0.002 | 0.020 |
|  | MBN | 1.500 | 0.553 |
|  | MBP | 2.791 | 0.257 |
|  | SWC | 6.410 | 0.852 |
|  | pH | 3.435 | 0.106 |
| Main N-fixing bacteria phylum | p__Chlorobi | 9.000 | 0.353 |
|  | p__Chlorophyta | 20.407 | 0.086 |
|  | p__Actinobacteria | 5.170 | 0.054 |
|  | p__Proteobacteria | 3.707 | 0.054 |
|  | p__Euryarchaeota | 6.536 | 0.427 |
|  | p__Bacteroidetes | 2.965 | 0.699 |
| N-fixing bacteria richness and diversity | Chao1 | 0.089 | 0.052 |
|  | Shannon | 1.940 | 0.003 |

Note: *P* < 0.05 in the table indicates that there is a significant difference in this index between gentle slopes and steep slopes.

**Supplementary Table 7** Simple Effects Results

| Name | Explains % | pseudo-*F* | *P*-value |
| --- | --- | --- | --- |
| TK | 14.9 | 5.2 | 0.002 |
| pH | 12.0 | 4.1 | 0.002 |
| Slope | 10.6 | 3.6 | 0.006 |
| AN | 7.3 | 2.4 | 0.108 |
| SOM | 7.0 | 2.2 | 0.06 |
| MBC | 6.6 | 2.1 | 0.05 |
| AP | 5.9 | 1.9 | 0.148 |
| MBP | 5.3 | 1.7 | 0.224 |
| TP | 4.7 | 1.5 | 0.182 |
| N-NO_3_^-^ | 4.6 | 1.4 | 0.206 |
| TN | 3.3 | 1.0 | 0.258 |
| AK | 2.3 | 0.7 | 0.584 |
| N-NH_4_^+^ | 2.3 | 0.7 | 0.418 |
| N level | 2.2 | 0.7 | 0.676 |
| SWC | 2.0 | 0.6 | 0.708 |
| MBN | 1.9 | 0.6 | 0.734 |
